# Subcutaneous and Visceral Adipose Tissue Secretions from Extremely Obese Men and Women both Acutely Suppress Muscle Insulin Signaling

**DOI:** 10.3390/ijms18050959

**Published:** 2017-05-02

**Authors:** Ousseynou Sarr, Rachel Joyce Strohm, Tara Lynn MacDonald, Nicholas Gaudio, John Kenneth Reed, Jules Foute-Nelong, David James Dyck, David Michael Mutch

**Affiliations:** 1 Department of Human Health and Nutritional Sciences, University of Guelph, Guelph, ON N1G 2W1, Canada; osarr@uoguelph.ca (O.S.); strohmr@mcmaster.ca (R.J.S.); tmacdo01@uoguelph.ca (T.L.M.); ngaudio54@gmail.com (N.G.); ddyck@uoguelph.ca (D.J.D.); 2 Guelph General Hospital, Guelph, ON N1E 4J4, Canada; jkrmd@bell.net (J.K.R.); foutenelong@yahoo.com (J.F.-N.)

**Keywords:** adipokines, muscle insulin resistance, white adipose tissue, crosstalk

## Abstract

Adipose tissue plays a key role in the development of type-2 diabetes via the secretion of adipokines. The current study investigated if secretion media derived from intact visceral (VAT) and subcutaneous (SAT) adipose tissues from extremely obese men and women differently suppressed insulin signaling in human skeletal myotubes derived from a healthy, non-diabetic male and female donor, respectively. Adipose tissue samples were collected from men and women during laparoscopic bariatric surgery. In general, secretion media collected from both SAT and VAT depots caused impaired insulin signaling in myotubes, independent of sex. In females, this was true regardless of the protein kinase B (Akt) phosphorylation site (Akt ^Thr308^ and Akt ^Ser473^) assessed (*p* < 0.01). In males, both SAT and VAT secretion media reduced Akt ^Thr308^ activation in insulin-stimulated myotubes compared to controls (*p* < 0.001); however, only the VAT secretion media impaired Akt ^Ser473^ phosphorylation. Independent of sex, 13 out of 18 detected cytokines, chemokines, and growth factors were more abundant in VAT versus SAT secretion media (*p* < 0.01). Both SAT and VAT secretion media from obese men and women acutely suppress insulin signaling in myotubes, despite different secretion profiles. We propose that this crosstalk model will help to extend our understanding of the interplay between adipose and muscle, as well as the pathogenesis of type-2 diabetes.

## 1. Introduction

Diabetes is a health problem of increasing concern, with 422 million adults aged over 18 years living globally with diabetes in 2014 [1]. Skeletal muscle accounts for 80% of total glucose disposal under insulin-stimulated conditions [2]. Reduced skeletal muscle glucose uptake due to insulin resistance is the primary defect in type 2 diabetes [3,4]. Recent studies have reported that adipose tissues from overweight or obese compared to normal-weight individuals have an altered secretory profile, resulting in the increased release of many adipokines and pro-inflammatory factors [5,6]. Furthermore, metabolically beneficial adipokines, such as leptin and adiponectin, are secreted in higher amounts from both lean and obese subcutaneous adipose tissue (SAT) [7,8], whereas pro-inflammatory adipokines such as monocyte chemotactic protein-1 (MCP-1), interleukin-8, and interleukin-6 are more highly secreted from both lean and obese visceral adipose tissues (VAT) [9,10]. It is believed that these secreted factors play an important role in the crosstalk between adipose tissue and muscle, and contribute to the etiology of muscle insulin resistance. However, it is still not known if regionally distinct adipose depots affect muscle insulin signaling differently in humans, and whether this varies between men and women.

To date, the few human adipose tissue-muscle crosstalk models reported in the literature have used isolated adipocytes [5,11]; however, evidence shows that the other resident cell-types in adipose tissue contribute to its overall secretion profile [10]. In the current study, we have established an alternative human adipose tissue-muscle crosstalk model to investigate the impact of intact VAT and SAT secreted factors on skeletal myotube insulin signaling. We believe that our approach circumvents some of the limitations of previous studies by examining adipose tissue-muscle crosstalk using intact adipose tissue organ culture (ATOC) to avoid any potential confounders of collagenase-based isolation techniques, while simultaneously preserving the cellular heterogeneity and corresponding secretion profile of adipose tissue. The objective of the present study was to investigate if secretion media derived from intact VAT and SAT from extremely obese men and women differently suppressed insulin signaling in human skeletal myotubes.

## 2. Results

### 2.1. Effect of Secretion Media on Protein Kinase B (Akt) Proteins in Human Myotubes

**Figure 1:**
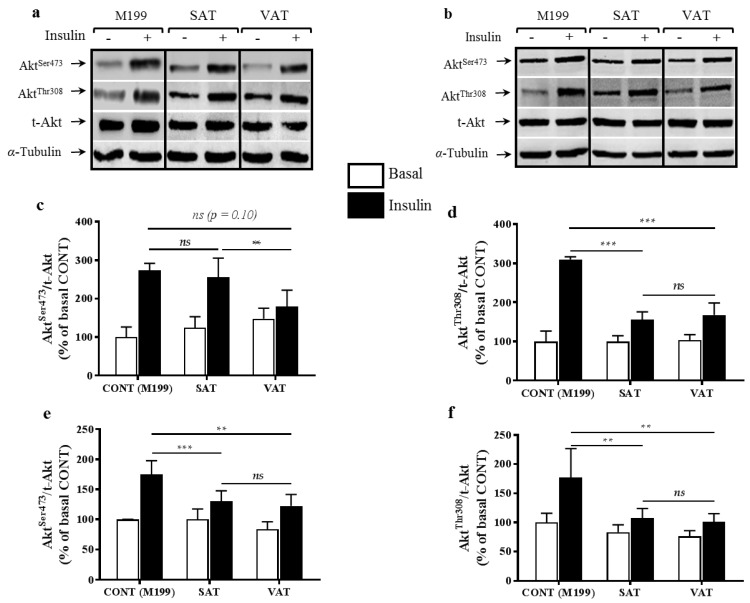
Human skeletal myotubes were incubated for 24 h with either control M199 or secretion media from visceral adipose tissue (VAT) or subcutaneous adipose tissue (SAT) of obese men and women. Myotubes were then stimulated acutely with insulin (100 µU/well, 10 min). Total cell lysates (20 µg/lane) were resolved by 10% sodium dodecyl sulfate-polyacrylamide gel electrophoresis (SDS-PAGE) and immunoblotted for total protein kinase B (t-Akt), Akt ^Ser473^, Akt ^Thr308^, and α-tubulin. Representative blots for muscle cells incubated with SAT and VAT media from one male donor (**a**) and one female donor (**b**) are shown. All phosphorylated Akt data were normalized to t-Akt and are expressed relative to the basal control (CONT) M199 value. Data are mean values ± standard error of the mean (SEM) of 10 different men (**c**,**d**) and 17 women (**e**,**f**) donors for SAT and VAT samples. **, ***, and “ns” indicate *p* < 0.01, *p* < 0.001, and not significant, respectively, for comparisons between insulin-stimulated conditions.

In males, both SAT and VAT secretion media reduced Akt ^Thr308^ activation (Figure 1a,d in insulin-stimulated myotubes compared to controls (*p* < 0.001). However, only the VAT secretion media impaired Akt ^Ser473^ phosphorylation (Figure 1a,c), although this was only significant when compared to SAT (*p* < 0.01) and not control (*p* = 0.104). Secretion media collected from female SAT and VAT caused significant reductions in Akt ^Thr308^ and Akt ^Ser473^ activation (Figure 1b,e,f) following insulin stimulation, compared to controls (*p* < 0.01). Secretion media from SAT or VAT for men or women did not affect total Akt (t-Akt) levels (Figure 1a,b).

### 2.2. Adipokine Content of Secretion Media

VAT and SAT secretion media was assessed to determine their adipokine profiles (Table 1). Eighteen out of 46 adipokines were detected in VAT and SAT secretion media. Independent of sex, 13 out of 18 detected adipokines were more abundant in VAT secretion media compared to SAT secretion media (*p* < 0.05). Only fibroblast growth factor-2 (FGF-2) was found to be more abundant in SAT secretion media compared to VAT secretion media (*p* < 0.001). No significant sex differences were observed for adipokine levels in VAT and SAT secretion media.

## 3. Discussion

We report here that, despite different cytokine profiles, both SAT and VAT secretion media from extremely obese men and women acutely suppress insulin signaling in myotubes to a similar extent. Moreover, we showed that VAT secretion media from obese men and women has higher levels of pro-inflammatory cytokines compared to SAT, which we attribute to differences in the tissue environment characterizing these two depots [12]. In addition, we demonstrate that utilizing intact adipose tissue, and not isolated adipocytes which ignore potentially important contributions from other cell-types, is an alternative approach to investigate adipose tissue-muscle crosstalk.

Co-culture of isolated adipocytes from normal-weight or obese individuals and primary human skeletal muscle cells from healthy donors have been reported during the last decade to study the crosstalk between adipose tissue and muscle in human [5,11]. While the use of isolated adipocytes has advanced our knowledge of adipocyte-myocyte crosstalk, collagenase-based isolation techniques may influence adipocyte function (e.g., reduced phosphodiesterase activity and insulin responsiveness) [13]. The effects of collagenase-based techniques on the adipocyte secretion profile are unknown. The current approach differs in that we have used intact adipose tissue from human subjects instead of isolated adipocytes. With this approach, the cellular heterogeneity of adipose tissue is preserved and no isolation techniques are used to extract a single cell-type. Moreover, using ATOC maintains the paracrine dialogue that exists between various cell-types within adipose tissue. Thus, we believe that the results obtained using our alternative human adipose tissue-muscle crosstalk model may provide more pathophysiological relevance than previous crosstalk models using isolated adipocytes.

A novel and key finding of the current study is the observation of an acute suppression of insulin signaling (reduced Akt ^Ser473^ and Akt ^Thr308^) in human skeletal myotubes incubated with SAT or VAT secretion media from obese men or women. An apparent difference in Akt phosphorylation sites in myotubes after incubation with the secretion media from men was also observed. However, at present, the precise mechanism leading to the differential regulation of Akt phosphorylation in male versus female myotubes warrants further investigation.

In the current study, 13 out of 18 detected adipokines were more abundant in VAT secretion media compared to SAT secretion media, independent of sex. Importantly, most of these adipokines act in concert in various inflammatory processes and are highly produced by adipose tissue with obesity and type-2 diabetes [14,15]. Furthermore, studies have shown that VAT has a greater pro-inflammatory profile compared to SAT, which has been linked to the development of insulin resistance [16,17]. However, despite most adipokines being more abundant in VAT secretion media, the effects on muscle insulin signaling were nearly identical for SAT in this study. A possible explanation for this somewhat unexpected finding may be related to the concentration of adipokines detected in the secretion media. In the current study, adipose tissue was obtained from extremely obese subjects (body mass index (BMI) ~50 kg/m^2^). The measured concentrations of some pro-inflammatory adipokines (e.g., IL-6 and IL-8, range of ~388–4861 pg/mL) in both SAT and VAT secretion media were extremely high relative to levels previously reported in the circulation (~8–43 pg/mL for IL-6 and ~4 pg/mL for IL-8; [18,19]), yet comparable to levels reported in adipose tissue secretion media (range of ~17–25 ng/mL/g of tissue; [20]) from less obese subjects. Nevertheless, given these high concentrations measured in the secretion media of both depots, it is tempting to speculate that this may have masked any subtle differences in terms of depot-specific effects on muscle insulin signaling. However, other adipokines (e.g. Regulated on activation, normal T cell expressed and secreted, RANTES) were present at very low levels in our secretion media (range of ~2–5 pg/mL) in comparison to circulating levels previously reported (14,600 pg/mL [21]), while MCP-1 was comparable to levels previously detected in plasma (~367–507 pg/mL [22]). Furthermore, the effect of these different adipokines on insulin signaling is unclear and may vary in relation to their concentrations. For example, only extremely high concentrations of IL-6 (250 ng/mL) and IL-8 (50 ng/mL) resulted in a slight impairment in insulin signaling in myotubes, whereas MCP-1 was only effective at concentrations similar to its physiological plasma level of 200 pg/mL [23]. Therefore, we acknowledge that some of the adipokines in SAT and VAT secretion media may be present at supra-physiological levels; however, other adipokines appear to be present at physiological and below physiological levels. Furthermore, the effects of specific adipokines on muscle insulin signaling may only be present at certain concentrations. Thus, whether or not the high concentrations of some adipokines observed in the present study in both SAT and VAT secretion media may be masking more subtle depot differences on myotube insulin signaling remains to be determined.

## 4. Materials and Methods

### 4.1. Characteristics of Muscle Cells and Donors of Adipose Tissues

Paired VAT and SAT samples were obtained from 13 men and 19 women undergoing laparoscopic bariatric surgery (Table S1) at the Guelph General Hospital (Guelph, ON, Canada). Ethical clearance was obtained from the Guelph General Hospital and the University of Guelph Ethics Boards (REB# 13JL020). Human skeletal myoblasts (HSkM-L, Cat. no. A11440) derived from a single healthy, non-diabetic male and female donor were purchased from Life Technologies (Burlington, ON, Canada).

### 4.2. Adipose Tissue Culture

Approximately 100 mg of VAT (epiploic) and SAT (periumbilical) was minced into small pieces and placed into wells of a six-well plate, containing 2 mL of oxygenated M199 media with 50 µU insulin (Humulin, Eli Lilly, Toronto, ON, Canada), 1.25 nmol/L dexamethasone, and 1% penicillin-streptomycin (P/S) (Sigma-Aldrich, Oakville, ON, Canada). Tissues were incubated at 37 °C in 5% CO_2_ for 24 h. After 24 h, media was changed and replaced with basic M199 media + 1% P/S. Twenty-four hours later, secretion media was collected and frozen at −80 °C for subsequent crosstalk analyses.

### 4.3. Determining Adipokine Content in Secretion Media

The secretion media profile from SAT and VAT was assessed using a ProcartaPlex panel (EPX450-12171-901, Fisher Scientific, Burlington, ON, Canada), in addition to adiponectin, interleukin-6 (IL-6) and tumor necrosis-α (TNF-α) panels (Fisher Scientific), and a Bio-Rad MagPix Multiplex Reader (Bio-Rad Laboratories, Mississauga, ON, Canada). Adipokine content was normalized to mg/mL of protein in SAT and VAT samples. Protein was quantified using a modified Lowry protocol, according to manufacturer’s instructions (Bio-Rad Laboratories).

### 4.4. Culture of Human Skeletal Myoblasts

Myoblasts were seeded in six-well plates at a density of ~960,000 cells per well and then differentiated using Dulbecco’s modified eagle medium (DMEM) (Fisher Scientific, Cat. no. 16050-122) supplemented with 2% horse serum (Life Technologies, Cat. no. 1693287). At day 5, all differentiated myotubes were switched to M199 media before being used for any experiment. After 24 h, M199 media was then replaced with VAT or SAT secretion media from ATOC studies (two wells per secretion media sample). Twenty-four hours post incubation with secretion media, one well was treated with 100 µU/well of insulin for 10 min. Therefore, each adipose depot per participant had a basal and insulin stimulated condition in myotubes. M199 media (with or without insulin) was used as the control for VAT or SAT secretion media (with or without insulin).

### 4.5. Immunoblotting

Following insulin treatment, proteins from myotubes were analyzed for the abundance of phosphorylated Akt ^Ser308^ and Akt ^Thr308^, *t*-Akt, and α-tubulin by directly lysing cells in nonyl phenoxypolyethoxylethanol (NP)-40 lysis buffer (Fisher Scientific) and following immunoblot protocols published previously [18].

### 4.6. Statistical Analysis

A non-parametric Mann-Whitney unpaired *t*-test was used to compare hemoglobin A1c (HbA1c) levels and BMI between men and women. All other data were analyzed using a two-way ANOVA and a Bonferroni post hoc test. For adipokine content in secretion media data, adipose tissue depot and sex were considered as main factors. For immunoblotting data, adipose tissue depot and treatment (basal vs. insulin-stimulated) were the main factors for each sex group. Data are reported as means ± SEM. *p* < 0.05 was considered statistically significant.

## 5. Conclusions

In summary, our results show that both SAT and VAT secretion media from obese men and women suppress insulin signaling in myotubes, and demonstrate that adipose tissue-muscle crosstalk can be studied in humans using an approach that does not require adipocyte isolation. As such, we believe this approach is suitable to investigate crosstalk between these two tissues in healthy and diseased states.

## Figures and Tables

**Table 1:** Proteins detected in visceral adipose tissue (VAT) and subcutaneous adipose tissue (SAT) secretion media from men and women.

Secreted Factor ^a,b,c^	Average in Male SAT	Average in Female SAT	Average in Male VAT	Average in Female VAT	SAT vs. VAT	Male vs. Female	Depot *x* Sex
IL-4	29.8 ± 7.2	40.6 ± 12.4	106.7 ± 13.3 ^†^	110.4 ± 23.3 **	< 0.001	0.689	0.846
IL-6	388.5 ± 72.1	699.6 ± 329.5	2183.6 ± 497.8	4861.1 ± 1590.1 **	0.004	0.141	0.242
IL-21	287.9 ± 15.6	285.3 ± 19.3	231.9 ± 13.1	213.8 ± 14.0 **	< 0.001	0.530	0.639
IL-1α	17.9 ± 0.9	18.4 ± 1.3	16.4 ± 0.7	15.6 ± 1.4	0.093	0.872	0.614
IL-1RA	65.7 ± 11.3	76.4 ± 18.9	79.2 ± 19.1	99.1 ± 19.9	0.349	0.427	0.813
GRO-α	50.2 ± 2.2	61.3 ± 9.0	125.3 ± 28.7	178.7 ± 54.6 *	0.008	0.358	0.546
IL-8	218.5 ± 61.3	586.9 ± 222.9	1427.5 ± 247.7	1866.5 ± 693.5	0.007	0.365	0.937
MCP-1	176.2 ± 55.2	234.3 ± 109.9	844.6 ± 150.3	949.8 ± 295.9 *	< 0.001	0.682	0.906
MIP-1α	10.6 ± 9.0	18.6 ± 39.3	29.9 ± 14.8	40.2 ± 48.4	0.033	0.332	0.901
MIP-1β	202.4 ± 11.8	214.1 ± 27.8	304.1 ± 16.9	297.0 ± 53.6	0.014	0.949	0.798
RANTES	1.9 ± 0.5	2.0 ± 0.6	4.6 ± 0.4 ^†^	4.7 ± 1.0 *	< 0.001	0.900	0.933
SDF-1α	314.2 ± 65.3	260.6 ± 79.6	622.2 ± 38.2	578.5 ± 114.6 *	0.001	0.593	0.956
FGF-2	87.3 ± 4.4	82.6 ± 6.0	70.4 ± 3.2	63.8 ± 4.6 *	< 0.001	0.262	0.852
HGF	694.3 ± 173.5	484.8 ± 101.3	950.7 ± 136.2	761.4 ± 140.8	0.058	0.1538	0.942
LIF	32.0 ± 7.0	55.4 ± 16.7	200.5 ± 32.1 ^‡^	213.6 ± 48.9 **	<0.001	0.589	0.879
P IGF-1	3.5 ± 0.7	2.5 ± 0.8	9.3 ± 1.6 ^†^	8.4 ± 1.8 **	<0.001	0.516	0.974
VEGF-A	117.3 ± 23.6	89.4 ± 14.5	253.6 ± 53.4 ^†^	169.9 ± 30.2	0.001	0.089	0.391
Adiponectin	3679.7 ± 648.2	3757.7 ± 565.4	3513.4 ± 426.5	3939.8 ± 610.0	0.989	0.670	0.769

^a^ Secreted factors were expressed in pg/mL per mg/mL of protein in VAT or SAT; Interleukin (IL)-4; IL-6; IL-21; IL-1α; IL-8; Interleukin-1 receptor antagonist (IL-1RA); Growth related oncogene alpha (GRO-α); Monocyte chemoattractant protein-1 (MCP-1); Macrophage inflammatory protein (MIP)-1α; MIP-1β; Regulated on Activation, normal T cell expressed and secreted (RANTES); stromal cell-derived factor -1α (SDF-1α); fibroblast growth factor-2 (FGF-2); Hepatocyte growth factor (HGF); Leukemia inhibitory factor (LIF); Placental growth factor-1 (P IGF-1); Vascular endothelial growth factor-A (VEGF-A); ^b^ Undetected adipokines: Brain-derived neurotrophic factor (BDNF); Eotaxin/C-C motif chemokine 11 (CCL11); Epidermal growth factor (EGF); Granulocyte-macrophage colony-stimulating factor (GM-CSF); Nerve growth factor)-β (NGF-β); Interferon (IFN)-α; IFN-γ; IL-1β; IL-2; IL-5; IL-7; IL-9; IL-10; IL-12 p70; IL-13; IL-15; IL-17A; IL-18; IL-22; IL-23; IL-27; IL-31; IP-10/chemokine (C-X-C motif) ligand 1 (CXCL10); Tumor necrosis (TNF)-α; TNF-β/Lymphotoxin-α (LTA); Platelet-derived growth factor-BB (PDGF-BB); Stem cell factor (SCF); VEGF-D; ^c^ Secreted factors were analyzed using a two-way ANOVA, with adipose tissue depot and sex as main effects. The interaction between depot and sex was also analyzed. *p* < 0.05 was considered significant. † and ‡ indicate *p* < 0.05 and *p* < 0.01, respectively, for comparisons between SAT and VAT in males after a Bonferroni post hoc test; * and ** indicate *p* < 0.05 and *p* < 0.01, respectively, for comparisons between SAT and VAT in females after a Bonferroni post hoc test.

## Supplementary Materials

Supplementary materials can be found at www.mdpi.com/1422-0067/18/5/959/s1.

## Abbreviations

Akt	Protein kinase B
MCP-1	Monocyte chemotactic protein-1
Ser	Serine
Thr	Threonine
TNF-α	Tumor necrosis-α
