# Supplementary material for: Subcutaneous and Visceral Adipose Tissue Secretions from Extremely Obese Men and Women both Acutely Suppress Muscle Insulin Signaling
Source: Int J Mol Sci. 2017 May 2;18(5):959. doi: 10.3390/ijms18050959 (PMC5454872; doi:10.3390/ijms18050959)
Supplement: Supplementary file 1 [file ijms-18-00959-s001.docx]

**Supplementary Table 1.** Characteristics of bariatric patients.

| **Variable** | **Men, *n* = 13** | **Women, *n* = 19** |
| --- | --- | --- |
| BMI (kg/m2) | 50.0 ± 2.1 | 46.9 ± 1.5 |
| Age (years) * | 53.5 ± 1.8 | 43.1 ± 1.7 |
| HbA1c (%) | 7.1 ± 0.4 | 6.6 ± 0.4 |

* = Statistically significant differences between men and women (*p* < 0.05).
